# Zika virus and host protein interactions for understanding molecular mechanisms of pathogenesis and therapeutic development

**DOI:** 10.3389/fcimb.2026.1754465

**Published:** 2026-02-04

**Authors:** Xiaodong Han, Jiansen Du, Wenhui Li, Shuqian Yang, Huamuzi Sun, Guihua Wang, Haolong Cong

**Affiliations:** 1 College of Life Sciences, Inner Mongolia Agriculture University, Hohhot, Inner Mongolia, China; 2 Qingdao International Travel Healthcare Center, Qingdao Customs District, Qingdao, China; 3 Chinese Academy of Inspection and Quarantine, Beijing, China

**Keywords:** antiviral drug development, flavivirus pathogenesis, viral entry receptors, virus-host protein interactions, Zika virus

## Abstract

Zika virus (ZIKV) causes severe neurological disease, including microcephaly and Guillain-Barré syndrome, through complex interactions with host cell proteins. This review synthesizes the 2015–2025 published literature on ZIKV-host protein interactions and their therapeutic targeting. ZIKV enters cells via multiple receptor pathways: adhesion receptors (DC-SIGN, Hsp70), high-affinity entry receptors (ITGB4, GRP78, NCAM1), internalization receptors (integrin αvβ5, sialic acid), and endosomal receptors (AXL, TIM-1, CD300a). Viral structural proteins direct virion assembly, while nonstructural proteins NS1–NS5 suppress immune responses, remodel cellular membranes, and dysregulate gene expression. NS5 uniquely suppresses neurodevelopmental genes and disrupts ciliary function through nuclear localization, directly driving microcephaly pathogenesis. Therapeutic strategies include receptor antagonists, protease inhibitors, and polymerase inhibitors. However, receptor redundancy, viral protein multifunctionality, and pregnancy safety constraints limit clinical translation. This review identifies ZIKV-host protein interactions as therapeutic targets and highlights barriers to drug development.

## 1 Introduction

Zika virus (ZIKV) is a mosquito-borne flavivirus first isolated in 1947 from a rhesus monkey in the Zika Forest, Uganda. For nearly six decades, ZIKV caused only sporadic infections with mild symptoms. The 2015–2016 outbreak in the Americas fundamentally altered this epidemiological pattern (Faria et al., 2016). ZIKV rapidly spread from Brazil throughout Central and South America, infecting millions and causing severe neurological complications. The outbreak revealed a devastating association between maternal ZIKV infection and congenital Zika syndrome, which is characterized by severe microcephaly, brain malformations, and neurodevelopmental abnormalities in newborns (Rasmussen et al., 2016). ZIKV infection also caused Guillain-Barré syndrome and other neurological complications in adults (Cao-Lormeau et al., 2016). In 2016, the World Health Organization declared the outbreak a Public Health Emergency of International Concern.

Zika virus causes disease by utilizing different host cell receptors for cellular entry and by establishing intricate interactions with host proteins following entry (Agrelli et al., 2019). These protein interactions enable ZIKV to infect diverse cell types, including those in the fetal brain and placenta, and to manipulate host pathways that support viral replication while suppressing immune responses. This capacity to exploit multiple entry routes and commandeer cellular machinery is central to ZIKV dissemination throughout the body and the ensuing neurological and developmental pathology. Understanding these interactions is critical for identifying novel therapeutic strategies and for limiting the severity and scope of ZIKV infection.

Targeting ZIKV-host protein interactions represents a highly promising antiviral strategy that offers considerable advantages over traditional direct-acting antivirals (Kumar et al., 2020). This approach reduces the likelihood of drug resistance because host proteins are genetically stable and do not mutate like viral genomes, and it provides broad-spectrum activity since many host factors are exploited by multiple flaviviruses. ZIKV depends extensively on host cellular machinery throughout its life cycle, creating numerous therapeutic intervention points from viral entry through replication and assembly. Entry can be blocked by targeting multiple entry receptors. Structural protein interactions can be disrupted to impair capsid function and block virion assembly. Nonstructural proteins offer additional targets (Aw et al., 2025; Qiao et al., 2025).

## 2 ZIKV receptors

**Figure 1:**
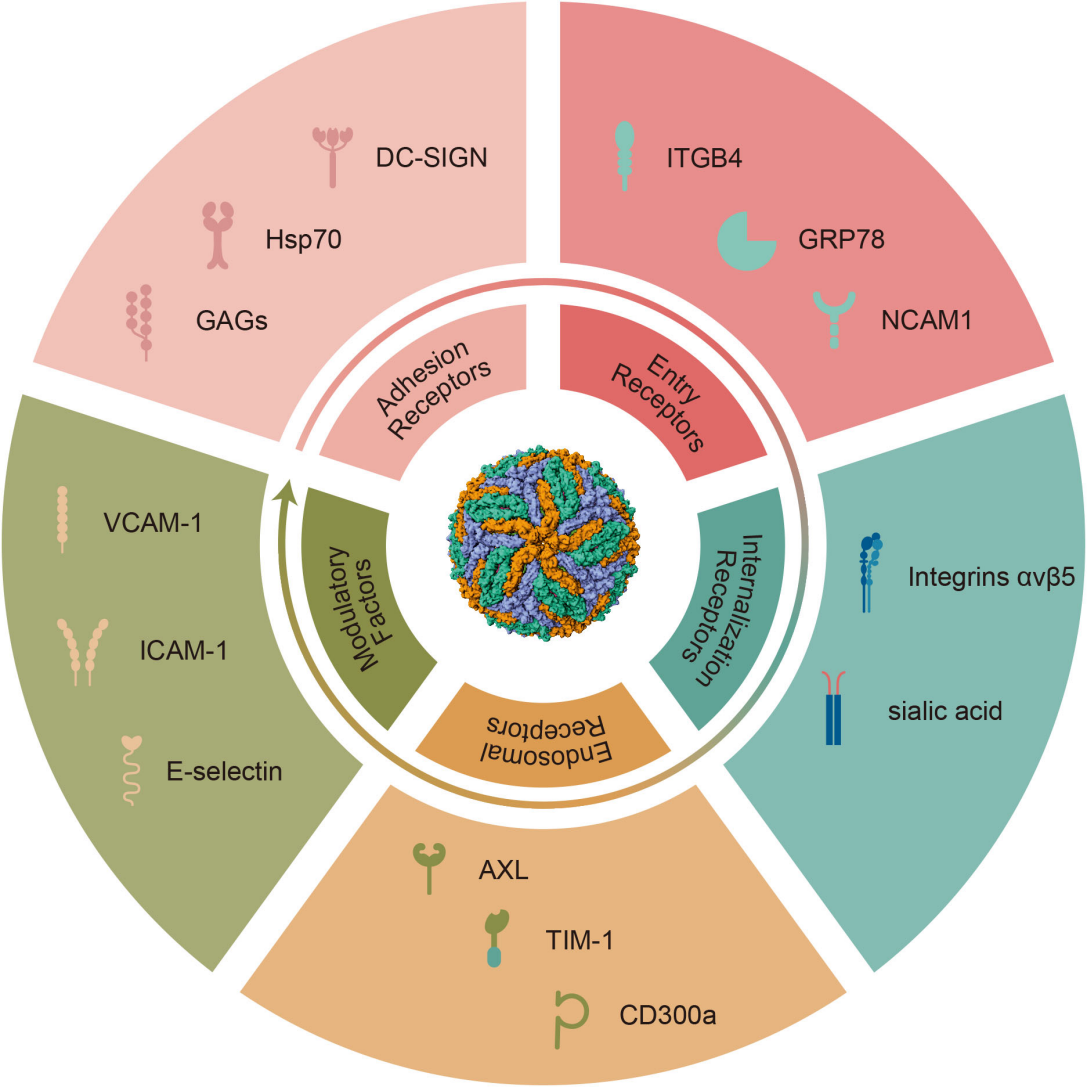
Multivalent ZIKV entry receptor network organized by functional class. ZIKV exploits hierarchical receptor classes for productive entry across diverse cell types. Adhesion Receptors (light pink) mediate initial low-affinity attachment via DC-SIGN, Hsp70, and GAGs. High-Affinity Entry Receptors (dark green) determine cellular tropism through ITGB4, GRP78, and NCAM1. Internalization Receptors (teal) facilitate endocytic uptake via integrin αvβ5 and sialic acid. Endosomal Receptors (coral) mediate pH-dependent membrane fusion through AXL, TIM-1, and CD300a. Modulatory Factors (ochre) including VCAM-1, ICAM-1, and E-selectin support immune cell trafficking. The central virion depicts E protein organization as the primary ligand for receptor engagement. This multi-receptor strategy enables neural tropism and explains vertical transmission through placental trophoblasts.

ZIKV infection proceeds through multiple, sequential stages involving diverse host receptors that perform distinct functional roles at different infection steps. This classification organizes confirmed and candidate ZIKV receptors according to their mechanistic contributions during viral entry and infection, from initial adhesion through membrane fusion (**Figure 1**).

### 2.1 Adhesion receptors

Adhesion receptors mediate initial, low-affinity interactions between viral particles and the cell surface, representing the first step of viral entry. Dysfunction of these receptors typically delays but does not eliminate infection, distinguishing them functionally from high-affinity entry receptors.

Dendritic Cell-Specific Intercellular adhesion molecule-3-Grabbing Non-integrin (DC-SIGN) is a C-type lectin receptor highly expressed on immature macrophages and dendritic cells. It serves as an attachment factor for multiple flaviviruses including DENV, WNV, JEV, and ZIKV, all of which display mannose-rich glycans recognized by the DC-SIGN (Singh et al., 2025). The N-terminus carbohydrate recognition domain (CRD) of DC-SIGN recognizes N-linked glycosylation sites on the ZIKV envelope protein in a calcium-dependent and mannose/fucose-selective manner (Menechino et al., 2023). This binding is specifically inhibited by anti-DC-SIGN antibodies, mannose competitors, or calcium chelators (EDTA).

Heat shock protein 70 (Hsp70) functions as a cell surface attachment factor facilitating ZIKV entry (Pujhari et al., 2019). Hsp70 translocates to the plasma membrane where it directly interacts with the ZIKV envelope protein. Hsp70 helps attach and concentrate ZIKV particles at the cell surface, and increase engagement with high-affinity entry receptors (Khachatoorian et al., 2018). Anti-Hsp70 antibodies significantly reduce ZIKV infection rates and plaque-forming units. Co-incubation of ZIKV with recombinant Hsp70 protein decreases ZIKV particle copy numbers in culture supernatants. Magnetic nanosensor studies further confirm direct binding interactions between Hsp70 and the ZIKV envelope protein (Shelby et al., 2017). Mechanistically, Hsp70 likely facilitates clathrin-mediated endocytosis, as Hsp70 localizes to clathrin-coated pits at the plasma membrane.

Glycosaminoglycans (GAGs) exhibit a paradoxical, context-dependent role in ZIKV infection that distinguishes it from related flaviviruses. Although ZIKV envelope protein binding pharmaceutical heparin with nanomolar affinity (KD = 443 nM for various sulfated GAGs) through electrostatically-driven, chain-length-dependent interactions with sulfated GAG motifs (Kim et al., 2017). However, multiple independent genome-wide CRISPR/Cas9 knockout screens definitively demonstrate that heparan sulfate (HS) biosynthesis gene deficiency (including EXT1, EXT2, EXTL3) does not significantly impair ZIKV attachment, internalization, or overall viral replication (Li Y. et al., 2019). This distinguishes ZIKV fundamentally from related flaviviruses such as DENV and JEV, which authentically depend on HS as obligate attachment co-receptors.​ It was proposed that HS promotes early viral replication by maintaining autophagy that ZIKV hijacks to establish replication organelles, but later restricts infection by targeting viral envelope and NS3 proteins for proteasome-dependent degradation (Ling et al., 2025).

### 2.2 High-affinity entry receptors

Following initial adhesion, entry receptors establish high-affinity binding between virus and cell surface, directly mediating productive viral entry. These receptors are the primary determinants of cellular tropism and cell-type-specific infection.

Integrin β4 (ITGB4, CD104) forms heterodimers with integrin α6 (ITGA6) on cell surfaces and serves as a critical entry receptor for ZIKV. ITGB4 demonstrates virus-specific selectivity. It mediates ZIKV entry but not JEV entry, distinguishing ZIKV from other flaviviruses that preferentially utilize integrin αvβ3. Soluble recombinant ITGB4 proteins and anti-ITGB4 antibody significantly reduced ZIKV binding and infection in a dose-dependent manner. *In vivo* studies further demonstrated that anti-ITGB4 antibodies prevented ZIKV infection in placental tissue, reduced viral titers in embryos, and increased embryo survival rates in pregnant mice (Li Y. et al., 2019).

Glucose-Regulated Protein 78 (GRP78) functions as a critical cell surface receptor facilitating ZIKV entry through direct binding to the ZIKV envelope protein domain III (EDIII) (Wang S. et al., 2020). GRP78 translocated from the ER lumen to the cell surface, where it serves as an attachment factor. The interaction involves both the nucleotide-binding domain (NBD) and substrate-binding domain (SBD) of GRP78, with critical residues (R492, T518) in the SBD mediating a canonical chaperone-client binding mechanism. Functional blocking experiments reveal that monoclonal antibodies targeting the N-terminus of GRP78 inhibit ZIKV entry by 50–70%, while siRNA-mediated GRP78 knockdown produces similar reductions. Following GRP78-mediated attachment, ZIKV enters cells predominantly through clathrin-mediated endocytosis, trafficking through Rab5^+^ early endosomes to Rab7^+^ late endosomes where low pH triggers E protein conformational changes and subsequent membrane fusion (Sanami et al., 2025).

Neural Cell Adhesion Molecule 1 (NCAM1/CD56) is an immunoglobulin superfamily glycoprotein highly expressed in brain tissue, neurons, glia, and skeletal muscle that functions as a high-affinity ZIKV entry receptor identified through time-resolved chemical proteomics (Srivastava et al., 2020). Multiple lines of evidence support this role, including direct binding of NCAM1 to the ZIKV envelope protein demonstrated via co-immunoprecipitation, blocking of ZIKV infection by the recombinant NCAM1 extracellular domain, reduced ZIKV infection upon anti-NCAM1 antibody treatment, enhanced ZIKV infection in NCAM1-overexpressing HEK 293T cells, and marked attenuation of ZIKV infection in CRISPR/Cas9-mediated NCAM1 knockout U-251 MG glioblastoma cells.

### 2.3 Internalization receptors

Internalization receptors specifically mediate viral internalization after virus-cell binding is established, rather than facilitating initial attachment. Integrin αvβ5 was identified through genome-wide CRISPR-Cas9 screening as a specialized receptor mediating ZIKV infection in neural stem cells (Zhu et al., 2020). Integrin αvβ5 specifically mediates ZIKV internalization rather than initial attachment (Wang S. et al., 2020). αvβ5 directly binds ZIKV virions and activates focal adhesion kinase (FAK), which is essential for productive viral infection. The expression level of αvβ5 correlates with ZIKV susceptibility in neural tissues and exhibits specific tropism patterns in the developing human cerebral cortex, with particularly high expression in neural stem cells. Notably, αvβ5-blocking antibodies and small-molecule inhibitors such as SB273005 and cilengitide effectively reduce ZIKV infection and alleviate virus-induced pathology in human neural stem cells and mouse brain tissue (Sanami et al., 2025).

Cell surface α2,3-linked sialic acid facilitates ZIKV internalization rather than attachment (Zhu et al., 2020). Neuraminidase treatment removing sialic acid significantly reduces ZIKV infection in Vero cells, neural progenitor cells, and Huh7 cells. Sialic acid is dispensable for viral attachment at 4°C during inactive endocytosis but essential for internalization at 37°C, demonstrating its role in endocytic uptake rather than binding. ZIKV does not directly interact with sialic acid, as sialyllactose fails to inhibit infection. Instead, sialic acid facilitates endocytosis-mediated entry. ZIKV specifically requires α2,3-linked rather than α2,6-linked sialic acid, as confirmed by linkage-specific neuraminidase treatments and ST3GAL4 knockout studies.

### 2.4 Endosomal receptors

Endosomal receptors localize to endosomal membranes and function under acidic pH conditions to mediate membrane fusion processes. AXL, a TAM receptor family member (TYRO3, AXL, MERTK), plays a complex and controversial role in ZIKV entry. Expression profiling showed positive correlation between AXL expression and ZIKV tropism in neural cells (Nowakowski et al., 2016). As a candidate receptor, AXL indirectly binds ZIKV via Gas6 and mediates viral entry through clathrin-mediated endocytosis to Rab5+ endosomes. The ZIKV/Gas6/AXL complex suppresses interferon signaling by activating AXL kinase, inhibiting ISG expression and type I interferon production, thereby facilitating ZIKV replication (Richard et al., 2017; Zwernik et al., 2021). However, AXL’s role as an essential ZIKV receptor remains controversial, while AXL knockout neural precursor cells remained susceptible to ZIKV infection (Wells et al., 2016). Pregnant mouse models revealed comparable ZIKV RNA levels across wild-type, AXL knockout, and MERTK knockout mice, indicating that AXL and MERTK are not essential for ZIKV infection in IFNAR-blocked mice (Hastings et al., 2017). Furthermore, a single PrM gene mutation (H83R) determines viral AXL-dependence, enabling AXL-independent infection of human neural cells and suggesting that AXL contributions vary by viral strain and cell type (Khasa et al., 2025).

Phosphatidylserine (PS) receptors play critical roles in late-stage ZIKV infection. T-cell immunoglobulin and mucin domain-containing protein 1 (TIM-1) has been confirmed as an important PS receptor enhancing ZIKV entry into cells. Cell electrical impedance biosensing studies demonstrate that ZIKV entry is highly sensitive to subtle changes in TIM-1 expression levels: both overexpression of TIM-1 in infection-resistant HEK293T cells and partial knockout of TIM-1 in susceptible A549 cells significantly modulate viral infection efficiency (Yu et al., 2025). Additionally, MARCH2 and MARCH3 function as redundant host restriction factors by targeting TIM-1 for K48-linked polyubiquitination and proteasomal degradation, thereby suppressing ZIKV infection (Zhang et al., 2025). CD300a represents another PS receptor recently confirmed to facilitate ZIKV cell entry (Oeyen et al., 2024). Inhibition of CD300a in immature monocyte-derived dendritic cells partially but significantly reduces ZIKV replication. Collectively, these findings demonstrate that ZIKV exploits multiple PS receptors to achieve efficient cell entry.

### 2.5 Modulatory factors

ZIKV infection of blood-brain barrier endothelial cells upregulates VCAM-1, ICAM-1, and E-selectin at mRNA and protein levels, with soluble forms released 4–10 days post-infection that facilitate leukocyte recruitment and adhesion to the BBB (Leda et al., 2019). Concurrently, ZIKV-infected primary human monocytes undergo proteomic reprogramming that upregulates integrins β1, α5, and αM; adhesion molecules ICAM-3, PECAM-1, CD99, and ITGAL; focal adhesion proteins including catenins, myosins, actinin, vinculin, talin, and filamins A/B; and the scaffolding protein IQGAP1 (Schouest et al., 2021). This adhesion molecule profile is ZIKV-specific, not replicated by HIV-1 infection or GM-CSF treatment, and functionally enhances monocyte attachment to endothelial cells and extracellular matrices. Coordinated upregulation of complementary adhesion molecules on both endothelial cells and infected monocytes, particularly CD14^+^ and CD16^+^ subsets, enables efficient monocyte transmigration across brain microvascular endothelial cells in transwell systems, with subsequent infection of basolateral astrocytes (Ayala-Nunez et al., 2019). While ZIKV utilizes multiple receptor pathways for cell entry, comparative analysis across flaviviruses reveals both conserved and virus-specific receptor usage patterns (**Table 1**).

**Table 1:** Comparative analysis of cellular receptor usage among flaviviruses.

Receptor/Entry factor	Evidence quality					
	ZIKV	DENV	WNV	JEV	YFV	TBEV
ITGB4	High	None	None	None	None	Not reported
ITGB3	None	Low	High	High	Not reported	Not reported
ITGAV/ITGB5	Medium	Not reported	Not reported	Not reported	Not reported	Not reported
ITGA6 with ITGB4	Medium	Not reported	Not reported	Not reported	Not reported	Not reported
Heparan Sulfate	Low	High	High	High	Medium	Medium
Syndecan (HSPG variant)	Not studied	High	Medium	Low	Not reported	Medium
AXL (TAM family)	Medium	Low	Low	Low	Low	Not reported
MER (TAM family)	Low	Medium	Medium	Medium	Medium	Not reported
TYRO3 (TAM family)	Low	Medium	Low	Not reported	Not reported	Not reported
TIM-1 (PS receptor)	Medium	High	High	Not reported	Not reported	Not reported
TIM-4 (PS receptor)	Medium	High	High	Not reported	Not reported	Not reported
DC-SIGN (C-type lectin)	Medium	High	Medium	High	High	Not reported
SIGN (C-type lectin)	Medium	High	Low	High	High	Not reported
GRP78 (Hsp90 family)	Medium	High	Medium	High	Medium	Medium
Hsp70 (Hsp family)	Low	Medium	Medium	High	Medium	Medium
NCAM1 (CAM)	Medium	Not reported	Not reported	Not reported	Not reported	Not reported
Claudin-1 (tight junction)	Low	Medium	Not reported	Not reported	Not reported	Not reported
CD55 (Complement)	Low	Low	Medium	Not reported	High	Not reported
Phosphatidylserine (lipid)	High	High	High	Medium	Medium	Not reported
Clathrin (endocytosis)	High	High	High	High	High	High

## 3 Structural proteins interacting with host proteins

### 3.1 ZIKV capsid protein

ZIKV capsid protein orchestrates viral assembly through extensive interactions with host cellular machinery. Quantitative proteomics in mosquito cells identified 157 host protein interactors, with the transitional ER ATPase TER94 and its human ortholog valosin-containing protein (VCP) (Gestuveo et al., 2021). TER94/VCP, together with E3 ubiquitin ligase UBR5, targets capsid for ubiquitin-proteasome-dependent degradation in both mosquito and human cells, and this paradoxical turnover is required for efficient infection (Giri et al., 2016). VCP inhibition markedly impairs viral replication and disrupts formation of replication organelles. Unlike dengue virus capsid, ZIKV capsid binds lipid droplets non-specifically but does not associate with very-low-density lipoproteins, linking capsid function to lipid-droplet-driven replication pathways (Qin et al., 2022).

### 3.2 ZIKV prM protein

ZIKV precursor membrane protein prM engages multiple host factors to regulate viral replication, virion assembly, and pathogenicity. PIM1 kinase directly binds and phosphorylates prM at Ser101 and Thr107, preventing AMFR-mediated ubiquitination and proteasomal degradation, stabilizing prM and enhancing viral replication (Ren Y. et al., 2024). The prM transmembrane domain contains two functional cholesterol-binding motifs, where CARC2 supports cholesterol-dependent viral entry and CARC3 promotes virion assembly. Disruption of either motif severely impairs infection (Goellner et al., 2023). prM carries an essential N-linked glycosylation site required for efficient virion secretion and infectivity (Gwon et al., 2020). The ER chaperone GRP78/BiP facilitates prM folding, while furin protease cleaves prM in the trans-Golgi network after pH-induced conformational changes, generating both mature and partially mature virions.

### 3.3 ZIKV E protein

ZIKV E protein is a class II fusion glycoprotein with three ectodomains: DI acts as a central scaffold, DII harbors the fusion loop and undergoes conformational rearrangement during fusion, and DIII mediates receptor engagement and tropism (Sevvana et al., 2018). A prominent N-linked glycan at Asn154 on the DI-DII hinge serves as an attachment site for DC-SIGN, which recognizes this glycan and mediates low-affinity initial adhesion (Sirohi et al., 2016). ZIKV E binds highly sulfated glycosaminoglycans such as heparin with nanomolar, electrostatic and chain−length−dependent affinity, supporting a role for GAGs as adhesion factors on placental and neural cells (Kim et al., 2019). Hsp70 translocates to the cell surface, binds ZIKV E protein, and functions as an attachment factor. GRP78 relocates to the cell surface, directly binds domain III of the ZIKV E ectodomain via its nucleotide−binding and substrate−binding domains, and promotes clathrin−mediated endocytosis and productive infection (Khongwichit et al., 2021). NCAM1 directly binds the ZIKV E ectodomain and acts as a high−affinity entry receptor in neural cells, with overexpression enhancing and knockout or blockade reducing viral binding and entry, thereby linking E–NCAM1 interaction to neural tropism and fetal brain infection (Srivastava et al., 2020).

## 4 Non-structural proteins interacting with host proteins

**Figure 2:**
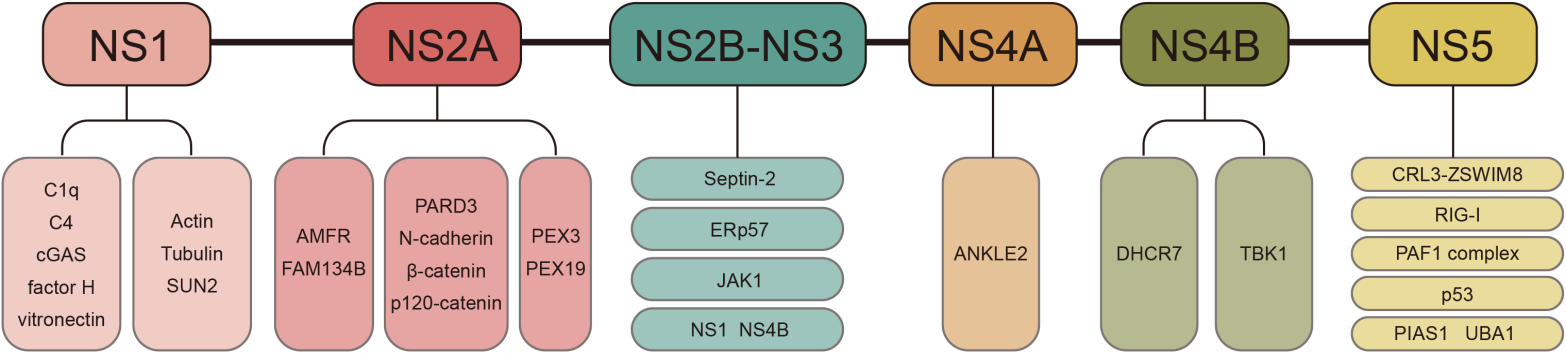
Virus-host protein interaction networks of ZIKV nonstructural proteins. The upper panel depicts sequential proteolytic cleavage generating NS1–NS5. The lower panel illustrates the host protein interactions for each nonstructural protein.

ZIKV nonstructural proteins NS1–NS5 are generated through sequential proteolytic cleavage of the viral polyprotein and orchestrate diverse pathogenic mechanisms through extensive host protein interactions (**Figure 2**).

### 4.1 NS1 protein

ZIKV NS1 orchestrates a multifaceted host interaction network comprising at least 28 proteins organized into five functional modules. The immune evasion module includes USP8, which NS1 recruits to stabilize caspase-1, facilitating cGAS cleavage and suppressing type I interferon responses, while secreted NS1 interacts with complement components C1q, C4, C4BP, factor H, and vitronectin to antagonize complement-mediated virolysis (Zheng et al., 2018). The chaperone module consists of the cytosolic chaperonin TRiC-CCT complex and ER chaperone GRP78, which provide ATP-dependent folding and stabilization of NS1 at ER replication sites (Wang Y. et al., 2020). The organellar module comprises mitochondrial proteins and ER lipid-associated factors that NS1 uses to remodel ER membranes into replication factories and siphon healthy mitochondria from neighboring cells via tunneling nanotubes (Ci et al., 2020). The cytoskeleton module includes actin, tubulin, and SUN2, which support tunneling nanotube formation and cytoskeletal reorganization required for NS1 trafficking and intercellular mitochondrial transfer. The stress response module involves RNase L and FMRP, RNA-binding proteins that NS1 co-opts to create a proviral environment (Michita et al., 2025). Together, these interactions enable NS1 to suppress interferon responses, establish ER-derived replication factories, promote intercellular dissemination through tunneling nanotube-mediated transfer, and reprogram host stress and RNA metabolism pathways to support viral replication.

### 4.2 NS2A protein

ZIKV NS2A functions as a multifaceted virulence factor orchestrating host protein interactions across several interconnected networks. The NS2A-AMFR-FAM134B axis suppresses ER-phagy through K48-linked ubiquitination at lysine 56, targeting RETREG1/FAM134B for proteasomal degradation and maintaining ER membrane supply for viral replication organelles (Zhang et al., 2024). This mechanism is evidenced by attenuated microcephalic phenotypes in ZIKV-NS2A K56R mutants that cannot undergo ubiquitination. NS2A also mediates STAT1/STAT2 degradation via a proteasome-dependent pathway involving amino acids 12-100, preventing nuclear translocation despite active phosphorylation and effectively suppressing interferon-mediated antiviral responses (Golubeva et al., 2020; Fanunza et al., 2021). In neural cells, NS2A disrupts adherens junction integrity through direct interactions with PARD3, CDH2/N-cadherin, CTNNB1/β-catenin, and CTNND1/p120-catenin, a mechanism uniquely absent in dengue virus NS2A that impairs neurogenesis and contributes to microcephaly in embryonic mouse cortex and human brain organoids (Kong et al., 2019). NS2A also manipulates peroxisomal biogenesis via PEX3/PEX19 interactions to reprogram cellular lipid metabolism, as ZIKV infection depletes peroxisomes in human fetal astrocytes while peroxisome expansion restricts viral replication (Wong et al., 2019). Furthermore, NS2A RNA directly binds eIF2α to suppress proinflammatory cytokine translation, adding a post-transcriptional layer to immune evasion (Wu et al., 2024). Collectively, NS2A maintains ER homeostasis for replication, evades interferon and NF-κB signaling, disrupts neural adherens junctions and neurogenesis, and reprograms peroxisome and translational pathways, positioning NS2A as a central orchestrator of ZIKV neuropathogenesis.

### 4.3 NS2B-NS3 complex

The ZIKV NS2B-NS3 serine protease complex interacts with host proteins to drive neurotoxicity and immune evasion. In neural progenitor cells, NS2B-NS3 directly binds and cleaves the cytoskeletal GTPase Septin-2 at residue R306, causing cytokinesis failure, supernumerary centrosomes, prolonged mitosis, and cell death that contribute to microcephaly pathogenesis (Li H. et al., 2019). NS2B-NS3 also interacts with the ER oxidoreductase ERp57, which stabilizes the NS2B/NS3 complex, enhances viral replication, and promotes reactive oxygen species-mediated DNA damage and apoptosis. ERp57 depletion reduces ZIKV production and limits DNA damage, highlighting this host factor as a proviral cofactor (Wang et al., 2024). Additionally, NS2B-NS3 promotes proteasome-dependent degradation of JAK1, blocking type I interferon signaling and reducing interferon-stimulated gene induction. This effect is reinforced when NS2B-NS3 cooperates with NS1 and NS4B to prevent interferon-induced autophagic degradation of the protease complex (Wu et al., 2017b). Proteome-wide interaction mapping identifies additional NS2B-NS3 host partners involved in cytoskeleton organization, vesicle trafficking, and cell cycle control, establishing this protease complex as a central hub that couples viral polyprotein processing to host cell remodeling, antiviral pathway suppression, and neural cell injury (Tangsongcharoen et al., 2019; Quek et al., 2022).

### 4.4 NS4A

NS4A is an ER-resident protein that remodels intracellular membranes by recruiting host factors to form replication organelles and support viral RNA synthesis. NS4A directly interacts with the microcephaly protein ANKLE2 and co-opts its role in coordinating nuclear envelope and ER organization (Fishburn et al., 2025). Loss of ANKLE2 or disruption of this complex impairs virus-induced membrane rearrangements and reduces ZIKV replication in mammalian and mosquito cells (Link et al., 2019). Genetic and functional studies show that NS4A-driven interference with ANKLE2-dependent pathways induces microcephaly-like phenotypes, linking this interaction to ZIKV-associated congenital brain malformations. Proteome-wide mapping indicates that NS4A also associates with ER membrane and stress-response factors involved in unfolded protein response signaling and ER homeostasis, consistent with the ER stress and UPR activation observed in ZIKV-infected neural progenitors (Shah et al., 2018). These findings establish NS4A as a membrane-remodeling hub that hijacks ANKLE2 and other ER-associated proteins to build replication factories, evade cellular stress surveillance, and contribute to ZIKV neurotropism and microcephaly.

### 4.5 NS4B

NS4B interacts with host proteins to remodel membranes and suppress innate immunity, promoting efficient viral replication and persistence. NS4B binds the cholesterol biosynthesis enzyme DHCR7 and induces its expression, which inhibits TBK1 and IRF3 phosphorylation, reduces IFN-β and interferon-stimulated gene induction, and enhances ZIKV infection in human cells and mouse models (Chen et al., 2023). Genetic or pharmacologic inhibition of DHCR7 restores antiviral signaling and restricts ZIKV replication. NS4B also blocks type I interferon induction by targeting the TBK1 signaling node, acting in parallel with NS1 and the NS2B-NS3 protease to dampen both interferon production and downstream JAK-STAT signaling (Sarratea et al., 2023). Systematic mapping places NS4B within ER-resident protein networks involved in membrane protein biogenesis and stress responses, consistent with its role in shaping replication organelles and adjusting ER homeostasis during infection (Han et al., 2021; Porter et al., 2025). These interactions establish NS4B as a multifunctional antagonist of interferon pathways and a coordinator of membrane remodeling via DHCR7-dependent and ER-associated mechanisms.

### 4.6 NS5

**Figure 3:**
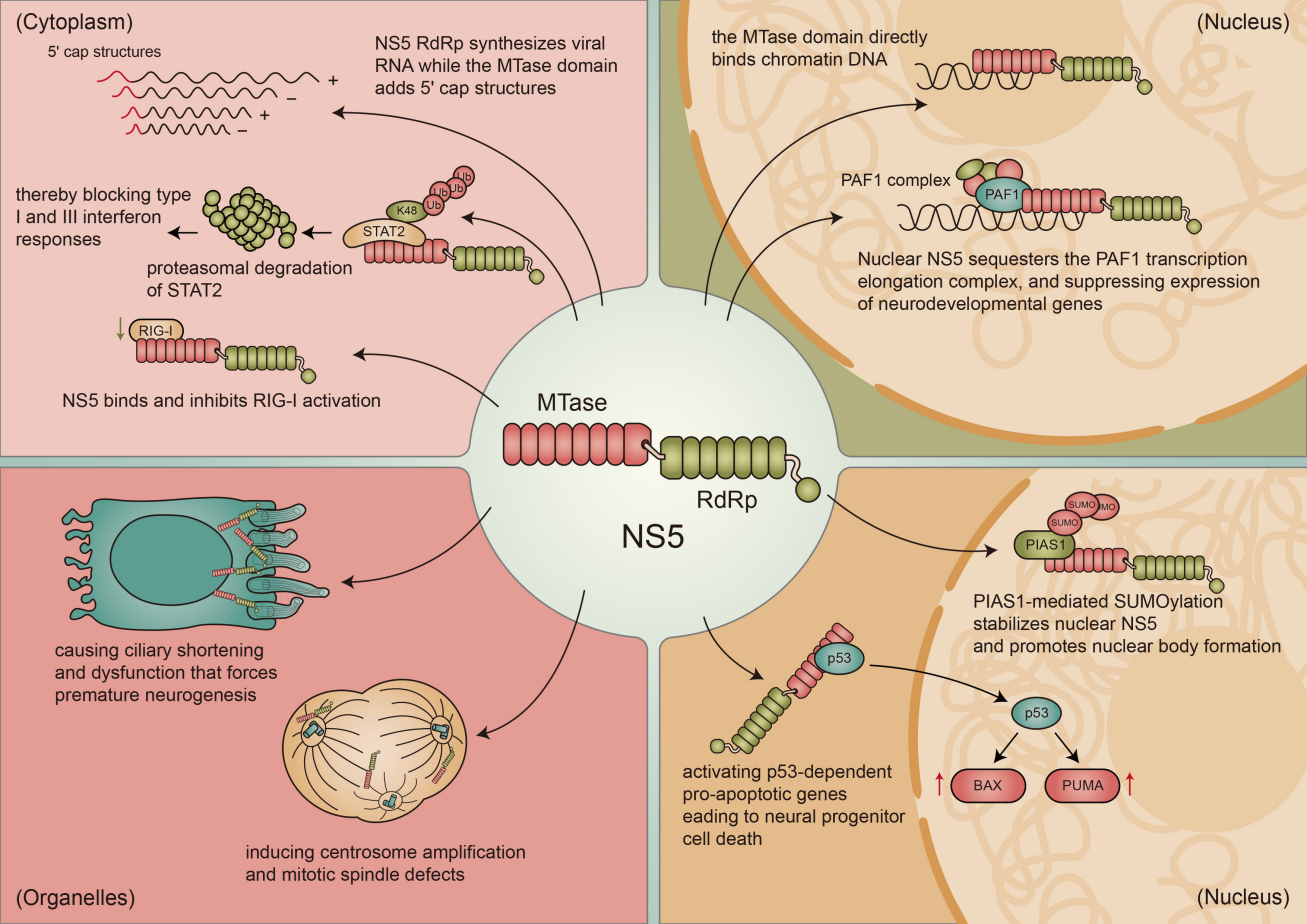
Compartmentalized mechanisms of NS5-mediated ZIKV neuropathogenesis. NS5 executes multiple pathogenic activities across cellular compartments. Cytoplasm (pink) NS5 RdRp generates viral RNA, recruits CRL3-ZSWIM8 for STAT2 degradation, inhibits RIG-I to suppress interferon, and multimerizes at cilia bases. Organelles (tan, lower left) NS5 promotes centrosome amplification and spindle defects. Nucleus (tan, right) NS5 suppresses neurodevelopmental gene transcription via PAF1 complex sequestration and is stabilized by PIAS1-mediated SUMOylation. Apoptosis (tan, lower right) NS5 activates p53-dependent BAX and PUMA transcription.

NS5 is the largest and most conserved flavivirus protein, containing an N-terminal methyltransferase domain and a C-terminal RNA-dependent RNA polymerase domain that support viral RNA capping and replication while engaging over fifty host proteins in multiple functional networks (Ferrero et al., 2019). Beyond its enzymatic roles, NS5 orchestrates immune evasion by promoting CRL3-ZSWIM8-dependent ubiquitination and proteasomal degradation of STAT2 to selectively block type I and type III interferon signaling while favoring type II interferon responses (Ren W. et al., 2024). NS5 also uses its methyltransferase domain to bind RIG-I and inhibit K63-linked ubiquitination and interferon-β production, and cooperates with viral sfRNA to inhibit STAT1 phosphorylation and downstream signaling (Li et al., 2020; Wu et al., 2022). Uniquely for a cytoplasmically replicating RNA virus, NS5 accumulates in the nucleus where it binds chromatin at actively transcribed neural genes and suppresses their expression by interfering with PAF1 complex-mediated transcription elongation, providing a direct mechanism for ZIKV-induced impairment of neurodevelopment (Li et al., 2022; Cai et al., 2023). NS5 multimerizes at the base of primary and motile cilia to induce non-genetic ciliopathy characterized by shortened or lost cilia, premature neurogenesis, abnormal neuron delamination, and ependymal ciliary dysfunction. NS5 also relocalizes to centrosomes during mitosis to promote centrosome amplification, spindle defects, and mitotic abnormalities in neural progenitors. Additionally, NS5 directly binds the C-terminal region of p53 to activate p53-dependent transcription of pro-apoptotic genes and trigger apoptosis in human neural progenitor cells (Li et al., 2021). NS5 stability is regulated by the SUMO E3 ligase PIAS1, which stabilizes flavivirus NS5 through SUMOylation, and by the ubiquitin-activating enzyme UBA1, which promotes ZIKV replication and modulates NS5 ubiquitin dynamics (Conde et al., 2020). NS5’s multifunctional activities spanning cytoplasmic replication, nuclear transcriptional repression, immune antagonism, centrosomal and ciliary disruption, and p53-driven apoptosis collectively explain its central role in ZIKV neurotropism and teratogenicity (**Figure 3**).

## 5 Drug research targeting Zika virus-host protein interactions

### 5.1 Antiviral drugs targeting Zikv-receptor interaction

Currently, there are no licensed antiviral vaccines or medicines for ZIKV infection. R428 (Bemcentinib) is an AXL tyrosine kinase inhibitor, demonstrating dose-dependent reduction of ZIKV infection in human fetal astrocytes and Sertoli cells (Meertens et al., 2017). The engineered soluble Axl decoy receptor MYD1 blocks infection by sequestering the bridging ligand Gas6, while polyclonal anti-Axl antibodies significantly reduce infection in endothelial cells and Sertoli cells (Meertens et al., 2017). CD300a inhibition targeting Phosphatidylserine receptor has validated as a therapeutic target, with in dendritic cells significantly reducing ZIKV replication (Oeyen et al., 2024). The lantibiotic peptide duramycin, which binds phosphatidylethanolamine and interferes with PS-mediated entry, inhibited ZIKV replication dose-dependently in astrocytes and Sertoli cells (Oeyen et al., 2021).

Small-molecule inhibitors targeting the envelope protein have been discovered using high-throughput screening methods—one study screened nearly 27,000 compounds and identified seven that directly block the Zika E protein, thereby preventing viral entry by disrupting its interaction with host cell receptors (Pitts et al., 2019; Telehany et al., 2020). Monoclonal antibodies that target unique epitopes on the E protein (including cryptic epitopes on domain III) have also shown potent neutralization, providing an additional strategy to inhibit virus–host attachment and entry (Wu et al., 2017a).

Broad-spectrum entry inhibitors include nanchangmycin, which blocks clathrin-mediated endocytosis and AXL-mediated entry at a pre-fusion stage; suramin, which prevents virus-cell surface binding; and plant-derived compounds such as Aphloia theiformis extracts that impair viral particle attachment. Neutralizing monoclonal antibodies targeting envelope protein epitopes—including C10, 2A10G6 (fusion loop), 5F8 (glycan loop providing full mouse protection), Z23, ZIKV-117 (reducing placental infection and maternal-fetal transmission)—have advanced to Phase 1 clinical trials.

13H10 monoclonal antibodies (anti-ITGB4) not only reduced ZIKV binding and infection *in vitro* but critically prevented placental infection, reduced embryonic viral titers, and increased embryo survival in pregnant mouse models, demonstrating robust protection against vertical transmission. Heat shock protein inhibitors targeting cell-surface Hsp70 (anti-Hsp70 antibodies and small-molecule inhibitor MKT077) reduced infection and protected mice from lethal ZIKV challenge without inducing resistance. GRP78/BiP antagonism via N-terminus-targeting monoclonal antibodies blocked entry and reduced infection by 50-70%, while siRNA knockdown confirmed its dual role in entry and replication factory formation (Khongwichit et al., 2021). NCAM1 blockade using NCAM1 extracellular domain recombinant protein or anti-NCAM1 antibodies significantly inhibited viral binding and entry, with CRISPR/Cas9 knockout dramatically reducing infection in glioblastoma cells. DC-SIGN antagonism through monoclonal antibodies and the carbohydrate-based blocker mannan prevented viral transmission to target cells. Neuraminidase treatment and genetic deletion of sialic acid biosynthesis genes (GNE, ST3GAL4) greatly reduced infection efficiency across multiple strains, validating the sialic acid pathway as a therapeutic target. Despite extensive preclinical validation, no receptor-targeted antiviral has reached late-stage clinical development, primarily due to receptor redundancy across tissues, cell-type-specific entry mechanisms, and the high safety bar required for pregnancy use.

### 5.2 Antiviral drugs targeting structural proteins-host proteins interaction

The FDA-approved VCP inhibitor CB-5083 impairs ZIKV infection in both human and mosquito cells and extends survival in flavivirus-infected mice (Anton et al., 2021). Additionally, SERTAD3 promotes capsid degradation, suggesting that enhancing this endogenous antiviral mechanism could suppress viral replication (Sun et al., 2023). Lipid droplet interactions are critical for capsid-mediated nucleocapsid assembly, and lipid metabolism inhibitors such as myriocin (a sphingolipid biosynthesis inhibitor) block ZIKV infection by disrupting lipid droplet-dependent particle assembly (Galilea et al., 2025).

Antiviral strategies targeting prM-host protein interactions work by disrupting key proviral pathways through three main mechanisms. First, inhibition of PIM1 kinase reduces phosphorylation of prM at Ser101 and Thr107, which restores AMFR-mediated ubiquitination and degradation of prM. Second, blockade of the cholesterol-binding motifs CARC2 and CARC3, or inhibition of upstream cholesterol biosynthesis, impairs both prM-dependent viral entry and virion assembly. Third, interference with prM folding and maturation through targeting of the ER chaperone GRP78/BiP or the furin protease prevents the cleavage of prM in the trans-Golgi network that normally generates infectious virions (Ren Y. et al., 2024).

### 5.3 Antiviral drugs targeting non-structural proteins-host proteins interaction

Monoclonal antibodies MAbs 3G2 and 4B8 target ZIKV NS1 and provide protection through Fcγ receptor dependent and independent mechanisms while avoiding antibody dependent enhancement (Yu et al., 2021). Regorafenib, a Raf kinase inhibitor, blocks viral translation, egress, and NS1 secretion to reduce pathogenesis (Wilken et al., 2024). Sofosbuvir, an FDA approved nucleoside analog polymerase inhibitor, suppresses NS5 replication in human cell lines and protects mice from lethal infection (Pagani et al., 2023). Galidesivir(BCX4430), a broad spectrum adenosine analog, abrogated viremia in rhesus macaques with favorable CNS penetration (Julander et al., 2021).

Proteasome inhibitors like MG132 block NS2A-mediated degradation of STAT1 and STAT2. This restores interferon signaling and antiviral immunity by directly targeting the NS2A-STAT pathway (Fanunza et al., 2021). NS3 protease inhibitors prevent proper NS2A maturation by blocking polyprotein processing. Theaflavin-3,3’-digallate is one example that indirectly reduces functional NS2A levels and its pathogenic host protein interactions (Cui et al., 2020). Sofosbuvir reduces overall nonstructural protein expression including NS2A. Raf kinase inhibitors such as Dabrafenib and Regorafenib impair ZIKV replication through post-entry mechanisms. These drugs suppress viral translation and egress and thereby limit NS2A-dependent virion assembly. AMFR E3 ubiquitin ligase inhibitors and FAM134B stabilizers represent emerging therapeutic candidates. These compounds could specifically block the NS2A K56 ubiquitination pathway to restore ER-phagy and prevent congenital pathogenesis.

Doxycycline binds the NS2B-NS3 active site and represents the only protease inhibitor clinically evaluated (Chong Teoh et al., 2021). Compound 71 demonstrates superior cellular efficacy as a competitive inhibitor awaiting clinical development. Allosteric inhibitors including SYC-1307, Compounds 1 and 2, temoporfin, and methylene blue target the protease’s “super-open” conformation (Coluccia et al., 2020; Meewan et al., 2023; Cavina et al., 2024). These drugs show potent activity in enzymatic assays and mouse models by preventing viral protein synthesis and brain damage. Peptide-based inhibitors like C-terminal hexapeptides containing YRRR motifs achieve high catalytic efficiency by occupying substrate-binding sites. Macrocyclic peptides incorporating d-lysine also demonstrate this mechanism (Nitsche et al., 2019).

Lipophilic statins block cholesterol biosynthesis to impair ZIKV replication that NS4B-DHCR7 interactions facilitate. Atorvastatin, cerivastatin, fluvastatin, lovastatin, mevastatin, and simvastatin reduce viral production by inhibiting HMG-CoA reductase (Espano et al., 2019). Ezetimibe inhibits cholesterol absorption and demonstrates synergistic effects with atorvastatin against ZIKV through complementary mechanisms (Osuna-Ramos et al., 2023). Avasimibe and other SOAT1 inhibitors block cholesterol esterification to impair ZIKV morphogenesis by accumulating free cholesterol and reducing extracellular viral RNA and envelope protein levels (Schobel et al., 2024). SBI-0090799 blocks *de novo* formation of membranous replication compartments by directly targeting the N-terminal region of NS4A (Riva et al., 2021). Dabrafenib and Regorafenib impair ZIKV replication through distinct post-entry mechanisms that affect NS4A and NS4B-dependent viral translation and egress (Wilken et al., 2024). PKI 14–22 suppresses ZIKV infection in endothelial cells and astrocytes by inhibiting protein kinase A without appreciable cytotoxicity (Cheng et al., 2018). AGC kinase inhibitors regulate STING signaling through SGK-dependent and independent mechanisms to counteract NS4B-mediated suppression of interferon responses (Betancur-Galvis et al., 2023).

Galidesivir (BCX4430) targets the NS5 RNA-dependent RNA polymerase domain and has completed Phase I clinical trials with demonstrated safety and favorable CNS penetration (Deshpande et al., 2023). Sofosbuvir inhibits NS5 polymerase activity and protects mice from lethal ZIKV infection while preventing vertical transmission in pregnancy models (Boccuto et al., 2021). Non-nucleoside RdRp inhibitors bind to the allosteric N pocket and priming loop sites on NS5 polymerase and demonstrate nanomolar potency against ZIKV replication (Aitken et al., 2025). AT-9010 binds both the GTP site of NS5 methyltransferase and acts as a chain terminator during RNA synthesis (Krejcova and Boura, 2025). SUMOylation inhibitors disrupt NS5 nuclear localization and impair formation of nuclear bodies required for persistent infection (Zhu et al., 2019). CB-5083 inhibits VCP protein function and disrupts NS5-dependent replication organelle formation in both human and mosquito cells (Anton et al., 2021). Suramin blocks NS5-NS3 protein interactions and prevents virus attachment to host cells with pan-flavivirus activity (Yang et al., 2022). The diverse therapeutic strategies targeting ZIKV-host protein interactions span from entry inhibition through replication suppression, with development stages ranging from preclinical evaluation to Phase I clinical trials (**Table 2**).

**Table 2:** ZIKV therapeutic targets and drug development status.

Drug	Viral or host target	Development status	Clinical trial stages
Small-molecule E inhibitors	E protein (E domain binding sites)	In vitro screening	None
Neutralizing mAbs (C10, 2A10G6, 5F8,Z23, ZIKV-117)	E protein (E protein epitopes)	Phase I clinical trial	Phase I (ongoing)
Nanchangmycin;Suramin	E protein entry	Preclinical in vitro/vivo	None
R428 (Bemcentinib), MYD1 (AXL decoy receptor); Anti-ITGB4 mAbs (13H10),Recombinant ITGB4; Anti-Hsp70 mAbs,MKT077; Anti-GRP78 mAbs, GRP78 siRNA; NCAM1 extracellular domain; Anti-DC-SIGN mAbs, Mannan carbohydrate; Neuraminidase/ST3GAL4 inhibitors	E protein (AXL pathway; ITGB4 pathway; Hsp70 pathway; GRP78 pathway; NCAM1 pathway; DC-SIGN pathway; sialic acid pathway)	Preclinical in vivo	None
CB-5083	Capsid (VCP ATPase)	Preclinical in vivo	None
Statins	prM (Cholesterol biosynthesis)	Preclinical in vitro/vivo	None
Proteasome inhibitors; AMFR/FAM134B stabilizers	NS2A (STAT1/STAT2 degradation; FAM134B ubiquitination)	Preclinical in vitro/vivo	None
Doxycycline	NS2B-NS3	Phase I clinical trial	Phase I
Compound 71; Allosteric inhibitors; Septin-2 stabilizers	NS2B-NS3 (NS3 protease active site; NS3 protease allosteric site; Septin-2 GTPase)	Preclinical in vitro/vivo	None
ANKLE2 stabilizers	NS4A (ANKLE2 ER protein)	Preclinical in vitro	None
DHCR7 inhibitors; PKI 14-22	NS4B (DHCR7 enzyme; TBK1 kinase)	Preclinical in vitro/vivo	None
Galidesivir (BCX4430)	NS5 (NS5 RdRp domain)	Phase I clinical trial (completed)	Phase I (completed 2024)
Sofosbuvir	NS5 (NS5 RdRp domain)	Preclinical in vitro/vivo	Preclinical
Non-nucleoside RdRp inhibitors; AT-9010; SUMOylation inhibitors; p53 activators	NS5(NS5 RdRp allosteric site; NS5 MTase domain; NS5 nuclear impor; p53 tumor suppressort)	Preclinical in vitro	None

## 6 Conclusion and future perspectives

ZIKV enters cells through sequential engagement of multiple receptor classes. ZIKV first binds adhesion receptors including DC-SIGN and Hsp70 for initial attachment, then utilizes high-affinity entry receptors such as ITGB4, GRP78, and NCAM1 that determine cell-type-specific infection. Internalization receptors including integrin αvβ5 and 2,3-linked sialic acid facilitate endocytic uptake, while endosomal receptors such as AXL and TIM-1 mediate low-pH fusion. This multi-receptor strategy enables ZIKV to productively infect neural progenitor cells, placental trophoblasts, and immune cells. Following entry, ZIKV structural proteins including capsid and prM direct virion assembly, while nonstructural proteins NS1-NS5 manipulate immune responses, endoplasmic reticulum architecture, cell-cell junctions, and transcriptional regulation. Although NS5 nuclear localization is shared among flaviviruses, ZIKV NS5 distinctly suppresses neurodevelopmental genes and causes ciliopathy through chromatin binding and PAF1 complex sequestration (Saade et al., 2020).

Identification of these virus-host interactions has revealed multiple therapeutic targets, including receptor antagonists, VCP and protease inhibitors, and NS5 polymerase inhibitors. However, clinical development faces obstacles: functional redundancy allows ZIKV to use alternative entry pathways when single receptors are blocked, multifunctional viral proteins compensate for inhibition of individual functions, and drugs must penetrate the blood-brain and placental barriers while meeting strict pregnancy safety standards. Combination strategies targeting multiple viral proteins simultaneously may overcome these challenges.

ZIKV-host protein interactions are largely conserved across flaviviruses including dengue, West Nile, and Japanese encephalitis viruses. Broad-spectrum antiflaviviral agents would address multiple viral threats, particularly as mosquito vector ranges expand with climate change. Advanced technologies including structural biology, CRISPR screening, and high-throughput screening provide tools for identifying and validating therapeutic targets. Comprehensive knowledge of virus-host interactions will enable rational design of therapeutics capable of preventing ZIKV-induced congenital disease and mitigating future flavivirus epidemics.
